# Towards an Ethological Animal Model of Depression? A Study on Horses

**DOI:** 10.1371/journal.pone.0039280

**Published:** 2012-06-28

**Authors:** Carole Fureix, Patrick Jego, Séverine Henry, Léa Lansade, Martine Hausberger

**Affiliations:** 1 Université de Rennes 1, UMR CNRS 6552 Ethologie Animale et Humaine, Rennes, France; 2 Université de Tours, Institut Français du Cheval et de l’Equitation, UMR 6175 INRA-CNRS Physiologie de la Reproduction et des Comportements, Nouzilly, France; Université Pierre et Marie Curie, France

## Abstract

**Background:**

Recent reviews question current animal models of depression and emphasise the need for ethological models of mood disorders based on animals living under natural conditions. Domestic horses encounter chronic stress, including potential stress at work, which can induce behavioural disorders (*e.g.* “apathy”). Our pioneering study evaluated the potential of domestic horses in their usual environment to become an ethological model of depression by testing this models’ face validity (*i.e.* behavioural similarity with descriptions of human depressive states).

**Methodology/Principal Findings:**

We observed the spontaneous behaviour of 59 working horses in their home environment, focusing on immobility bouts of apparent unresponsiveness when horses displayed an atypical posture (termed *withdrawn* hereafter), evaluated their responsiveness to their environment and their anxiety levels, and analysed cortisol levels. Twenty-four percent of the horses presented the withdrawn posture, also characterized by gaze, head and ears fixity, a profile that suggests a spontaneous expression of “behavioural despair”. When compared with control “non-withdrawn” horses from the same stable, withdrawn horses appeared more indifferent to environmental stimuli in their home environment but reacted more emotionally in more challenging situations. They exhibited lower plasma cortisol levels. Withdrawn horses all belonged to the same breed and females were over-represented.

**Conclusions/Significance:**

Horse might be a useful potential candidate for an animal model of depression. Face validity of this model appeared good, and potential genetic input and high prevalence of these disorders in females add to the convergence. At a time when current animal models of depression are questioned and the need for novel models is expressed, this study suggests that novel models and biomarkers could emerge from ethological approaches in home environments.

## Introduction

Depression is a major mood disorder with a high prevalence of morbidity (especially in women) and a heritable component. Severe forms of depression occur in 2–5% of the US population [1], [2]. Early ethological models of depression were based on observations of socially-deprived young monkeys and chicks that, after a protestation phase, gave way to “behavioural despair” by becoming hypoactive, silent and unresponsive to their environment *^e.g.^* [3]. These models have been replaced by more rapid and repeatable tests with rodents, such as the Porsolt and the tail suspension tests, where animals are considered to be depressed when they stop “resisting” *^e.g.^* [2], [4].

However, nowadays most reviews converge in questioning current animal models of depression [2], [5], [6]. After decades of using well-controlled animal models, the outcome however is a discrepancy between the positive influence of some drugs on these models and the lack of their efficiency for humans [7]. Nestler *et al.* [2] emphasized the need for better animal models and for biomarkers of depression and they regretted that none were available. They concluded that at that time no *bona fide* animal model of biomarker of depression existed, and that the researches involving animal models of mood disorders desperately needed a “focus on ethologically informed models of mood disorders with animals living under more natural conditions”, *i.e.* models focused on observing animals’ spontaneous behaviour in their home environments. A first step towards validating an ethological model is to insure its “face validity”, that is a comparability of symptoms, such as loss of interest in the environment, behavioural despair, apathy and possible anxiety-related behaviour [4]. Depression and anxiety may be associated and correspond to a continuum [8].

Ethological animal models may help to address the area of peripheral markers of depression, as for example increased or decreased cortisol levels in community/hospital groups of patients have been debated ([8]–[10], see also [11] in animals), and they should be useful to test aetiology and genetic components of this disorder. Animal emotionality is considered a reliable model of human anxiety and has a heritable component [12]. Despite recent attempts to develop novel models (*e.g.* guinea pigs’ isolation-induced calling paradigm or gerbils’ foot tapping response, [6]) no satisfactory animal model has emerged. Ethological approaches, focused on animals’ spontaneous behaviour in their home environments, might prove to be useful especially if the environmental conditions offered to animals share features with environmental conditions known to induce depression in human. For instance, stress at work, and especially interpersonal stressors, may lead to a variety of negative and durable effects, such as depression [13]. Domestic horses may encounter social and spatial restriction (as several domestic or lab species) but also share with humans the characteristic of working on a daily basis and have then ‘‘interpersonal’’ interactions (with other working horses, working with a ‘‘boss’’ who is the human who manages or rides it) [14]. Horses’ studies, mimicking in animals conditions in which human depression is known to occur, might then open a “window” onto the complex aetiology of human depression.

According to recent studies (though not designed in order to compare collected data with human depression characteristics), “apathy”, “withdrawal” and/or “inhibition” can occur in sows, pigs, sheep or dogs submitted to social/spatial restrictions or to repeated aversive stimuli inducing chronic stress [15]–[17]. Recently, two studies respectively suggested or reported the occurrence of “apathetic”, “unresponsive”, “switched off” horses in a restricted domestic environment [18] or under harsh conditions in developing countries [19]. Previous observations performed by the authors in horses from riding school also revealed that part of this population appears to display an atypical posture (standing motionless with eyes open, stretched neck and similar height between neck and back), when horses seem to display an apparent unresponsiveness, *i.e.* seem to have “withdrawn”. In the present study, we hypothesized that this symptomatology in working horses, termed *withdrawn* hereafter, could suggest human depression characteristics. We tested this hypothesis by observing horses’ spontaneous behaviour in their home environment, evaluating their reactivity to their environment and by taking physiological samples (plasma cortisol levels assessment). This pioneering study aims to evaluate the potential of domestic horses in their usual environment to become a potential ethological model by testing this model’s face validity, before performing further more invasive studies. We focused on two elements: spontaneous expression of “behavioural despair” and unresponsiveness to a variety of environmental stimuli (tactile/visual, human/non-human).

## Materials and Methods

All experiments comply with current French laws (Centre National de la Recherche Scientifique) related to animal experimentation and were in accordance to the European Communities Council Directives of 24 November 1986 (86/609/EEC). Mostly behavioural observations were performed. Total duration of the blood sampling procedure did not exceed 1 min, and non-painful sampling was confirmed by the absence of any retreat behaviour of the horses during the procedure. Animal husbandry and care were under management of the riding schools staff, as this experiment involved only horses “from the field” (no laboratory animals).

### Subjects

Fifty-nine horses from three riding schools were observed between January and June 2007 (**table 1**). Activities and housing conditions in the schools were similar. In all cases, the horses were kept singly in 3 m * 3 m individual straw-bedded boxes. Each box was cleaned once a day (in the morning). Animals were fed commercial pellets three times a day and hay was provided *ad libitum* once a day. Each box was equipped with an automatic drinker. Horses worked in riding lessons for 4–12 hours a week, with at least 1 free day each week (closing day). Riding lessons involved children and teenagers and were mainly related to indoor (instruction) and outdoor activities, including a few competition activities. Both geldings (n = 44) and mares (n = 15) were tested. Sixty-eight percent of the horses were French Saddlebreds, equally distributed among the centres. Other horses belonged to a variety of breeds or were unregistered animals. They were between 5 and 20 years old (median  = 12, 1^st^ quartile  = 9, 3^rd^ quartile  = 14).

**Table 1:** Study population: horses’ breed, sex and age for each riding school.

	School 1	School 2	School 3
*Breed*			
French Saddlebred	6	25	9
Connemara	1	–	–
French Trotter	1	1	1
Thoroughbred	1	–	–
French pony	1	–	–
Hanoverian	–	1	–
Anglo-Arab	–	1	–
Criolo	–	–	1
Iberian	–	–	1
Unregistered	2	1	6
*Sex*			
Mare	4	6	5
Gelding	8	23	13
*Age*			
mean ± errorstandard (range)	12.6±3.1 (8–17)	11.9±2.5 (6–16)	11.2±4.9 (5–20)

### Behavioural Observations in the Box

**Figure 1:**
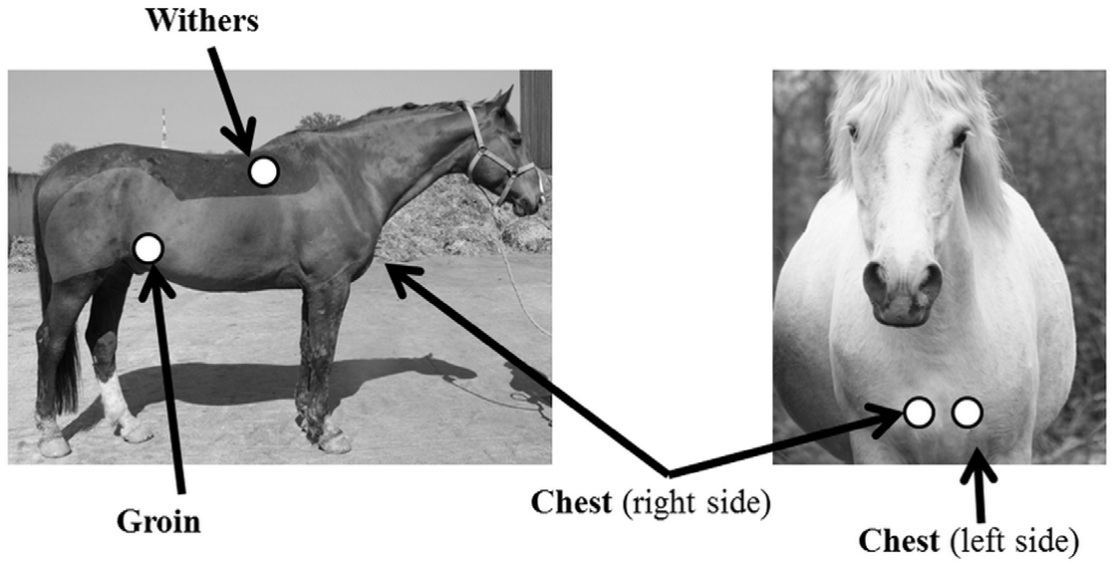
The withdrawn posture of “withdrawn” horses. Pictures of a horse a) in a withdrawn posture, b) standing non-resting and c) resting. Withdrawn posture is characterized by a similar height between the horse’s neck and back (the nape – withers – back angle approximately 180°) and a stretched neck (obtuse jaw-neck angle). This posture is distinguished from postures associated with observation of the environment (for which the neck is higher), and resting, when eyes are at least partly closed and the horse’s neck is rounder [20], [21]. Note that the restricted size of the box (3 m * 3 m) prevented the authors from taking a picture of the whole horse displaying the withdrawn posture, as we chose to use the same lens in order to limit shape distortion between pictures.

Horses were observed in their box using a focal sampling method: all behaviours of the focal animal were recorded continuously during 5-min sessions. Observations were made during three periods: 9–11 a.m., 2–5 p.m. and half an hour before meals (*i.e.* between 6.30–7.30 a.m., 11.30–12.00 a.m. or 5.30–6.00 p.m. according to riding school schedule). All horses were observed 6 times (2 sessions per period  = 30 min total/horse). All observations were made by a single observer. All behaviours were recorded and special attention was given to withdrawn bouts, *i.e.* bouts of immobility when horses displayed a unusual posture, characterized by horses standing with eyes open, stretched neck (open jaw-neck angle) and similar height between neck and back (**Fig.1**). During observations, this posture was easily distinguished from observation of the environment, when the horse’s neck is held higher [20], and from resting, when “in the standing posture, the horse is supported usually by only 3 legs with the slope of the neck lower and rounder than when attentive and alert. The muscles relax, the ears rotate laterally, the eyelids and lips get droopy and the eyes close. In the extreme, the crest of the neck may drop 20° or more below horizontal, with the dorsal surface of the head sometimes reaching vertical” ([21], see also [20] for objective inter-behavioural comparisons of postures).

“Apathetic”, “unresponsive”, “switched off” horses” are described as having “dull eyes looking nowhere” [18], [19]and therefore special attention was paid to eye and ear fixity (and position) when horses displayed the withdrawn posture. Ear position is an interesting indicator of a horse’s internal state [21]–[23]and welfare (Fureix *et al.* in prep) and we have defined positions by referring to studies of other species [24]: axial ear (perpendicular to the head – rump axis), forward ear (tip of the ear towards the front at an angle of more than 30° from the perpendicular) or backward ear (tip of the ear towards the back at more than 30° from the perpendicular). Discomfort, pain and stress appear to be associated with ears in a backward position [21]–[23], Fureix *et al.* in prep]. Head movements, gaze durations and ears movements during these bouts were therefore compared to those of four other non-working horses (two geldings and two stallions, 13–20 years old) living under natural conditions (*e.g.* not socially deprived, not confined, not regularly exercised) and observed for 30 min each while standing non resting.

### Testing Responsiveness to Environment

#### 1) To tactile stimulations

Reactions to tactile stimuli are common in horses as an adaptive response to the presence of insects such as flies. Horses react by a muscular twitching [25]. Here we estimated tactile reactivity using von Frey filaments (Stoelting, IL, USA), a procedure first used by Redua et al. [26]on horses and adapted by Lansade *et al.* [27]. A filament consisted of a hard plastic body extended by a nylon thread and is calibrated to exert a specific force on the skin (namely 0.008, 0.02, 1 and 300 g/matter in our study). They were applied perpendicularly on an animal’s skin until the nylon thread started to bend. Muscular twitching (reaction used by horses to drive away flies [25]), was recorded.

**Figure 2:**
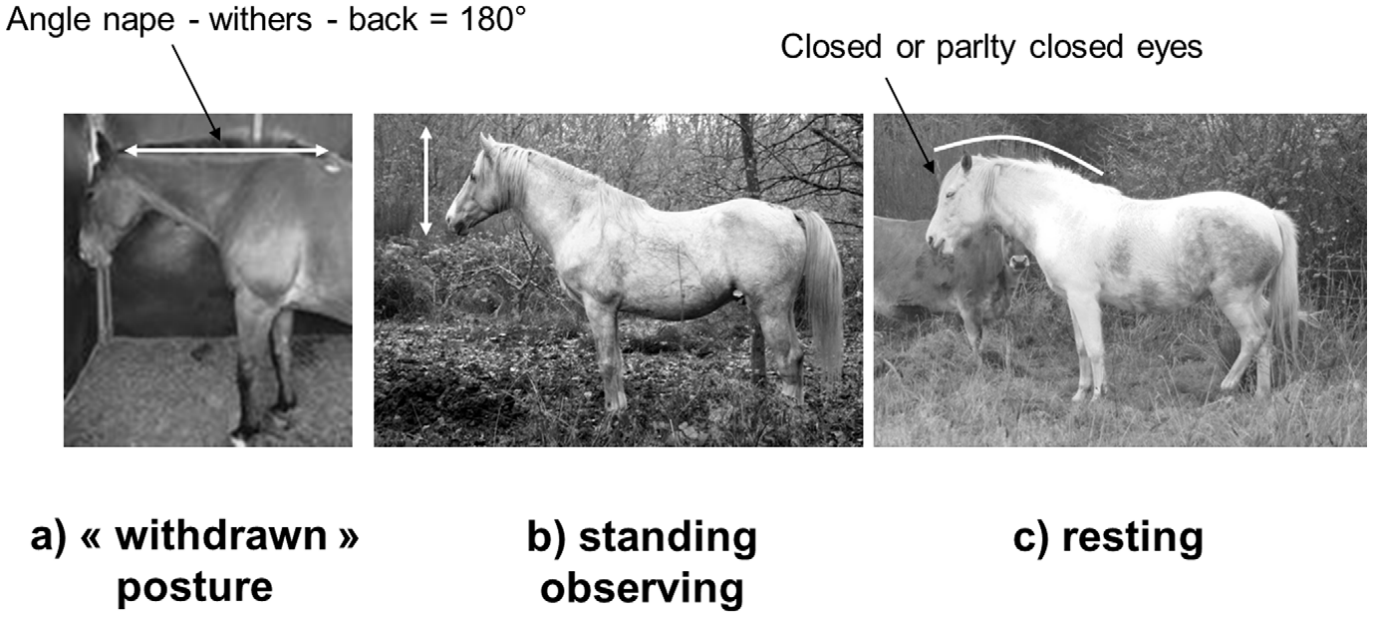
Test areas for tactile sensitivity assessment: withers, chest and groin.

Sensory reactivity was tested by the same unfamiliar experimenter (blind to the result of behavioural observations) in each individual box outside horses’ working times. The horse was lightly restrained and von Frey filaments were applied at the basis of the horse’s withers, groin and chest. Both sides of the horse were tested. Von Frey filament (0.008, 0.02, 1 and 300 g/matter) types, areas (withers, groin and chest, **Fig. 2**) and sides were tested in a random order for each horse. The tests involved two sessions (separated by 2 hours) during which two von Frey filaments were tested at 10 minutes interval. For instance, following a pre-established random order, a given horse was tested first at 08:30 p.m. using the 0.02 and 300 g/matter von Frey filaments, then re-tested at 10:30 p.m. using the other von Frey filaments (namely 0.008 and 1 g/matter in this example). The response was coded in a binary form (trembling/no trembling), in accordance with Lansade *et al.* [27].

#### 2) To human approach

Horses may be surprised by the sudden appearance of a human at their box door and their reactions can then vary from friendly approach to indifference or even aggression [28]. A previous study performed in the same 59 horses revealed inter-individual variations in the horses’ responses to a sudden human approach: *e.g.* 12% of them reacted friendly at least once, while 51% of them displayed at least one aggressive reaction [29], strongly suggesting that, even if horses from riding school might be habituated to people suddenly appearing at the door of the box, a high proportion of them still reacts (either negatively or positively) to the situation. We hypothesised that “depressed” horses would be indifferent to such a stimulation. The experimenter, walking slowly along the corridor, appeared suddenly at the closed door of the box while the horse was feeding (hay, straw), head down. She recorded the horse’s first reaction, following Hausberger and Muller’s [28] scoring method. Thus, five scores ranged from very ‘‘friendly’’ to very aggressive behaviour: the horse looks at the experimenter with upright ears and approaches: A; the horse looks at the experimenter with upright ears and remains where it is: B; the horse shows no evidence of directed attention towards the experimenter (no change in behaviour, no gaze towards the person): C; the horse looks at the experimenter with ears laid back and remains where it is: D; the horse looks at the experimenter with ears laid back and approaches with a threatening posture (neck lowered, head extended or even exposed incisors): E. Each horse was tested five times, at different times of the day, yielding five scores for each horse for each test. For instance, a given horse could score “C” in the first, second and third sudden approach tests, “B” in the fourth test and “C” again in the last test. This would yield five scores: CCCBC for that horse.

**Figure 3:**
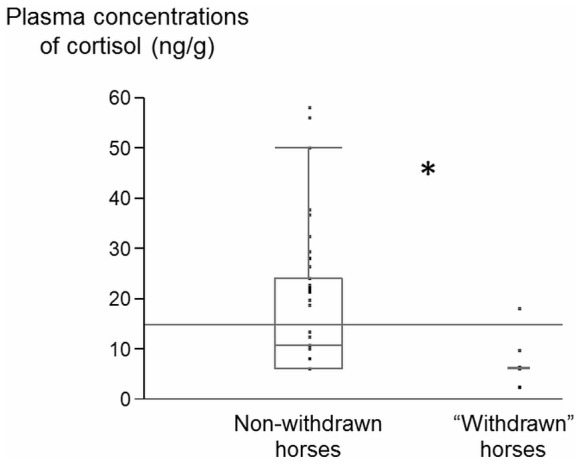
Behavioural characteristics of “withdrawn” bouts of “withdrawn” horses compared to horses standing non-resting observed under natural conditions. (a) rates of ear position changes (number of ear position changes per number of withdrawn/standing non-resting bouts); (b) rates of head position changes (number of head position changes per number of withdrawn/standing non-resting bouts, and (c) gaze durations of withdrawn/standing non-resting bouts (s) for “withdrawn” horses and horses standing non-resting observed under natural conditions. Data are given as boxplot diagrams showing medians (lines in the boxes), 25 and 75% quartiles (boxes) and minimum and maximum ranges (whiskers). Unusual features of “withdrawn” horses are the absence of ear and head movements and a fixed gaze.

### Are “Depressed” Horses also more Anxious?

Reactions towards a novel object while being released in a large environment (arena) are considered to reflect the horses’ level of emotionality [30] and more precisely nervousness [31]or anxiety[32]–[34]. Our procedure and measures were the same as Wolff et al.’s [30](see also [31], [32], [34]. A novel object, *i.e.* a cage formed by metallic rails and long red fluorescent ribbons (100 * 80 * 80 cm), was placed in a familiar arena where the horse was released for 5 min. As in the above cited studies, the horse’s behaviour was recorded by a motionless observer using instantaneous scan sampling [35] every 10s. The behavioural patterns sampled were: (a) standing; (b) exploration (characteristic slow walk of a quiet horse in a calm situation), (c) sustained walk, (d) trot, (e) passage, (f) gallop, (g) vigilance and (h) tail posture (detailed in *e.g.* [30]). Rare or brief behavioural patterns such as snorts, pawing, defecation, rolling, whinnying were recorded *ad libitum*. To rank the reactivity of horses, we used an emotionality index based both on behavioural patterns and their frequencies of occurrence; this index proved useful in previous studies. Values were attributed to the behavioural patterns according to their degree of specificity and corresponding level of arousal. These values were exploration  = 1, sustained walk  = 2, trot or gallop  = 3, vigilance  = 4 and passage, snorting or tail raised  = 6 (“5” was used in another test assessing reaction to social separation and is whinnying, not scored here). These values were multiplied by the number of times the corresponding pattern was observed. Note that these values only give a ranking indication and do not represent data on ratio scale (a horse with an index twice as high as that of another horse was not necessarily twice as reactive).

### Physiological Data: Cortisol Measures

Horses’ cortisol levels were assessed by blood sampling. In order to minimise the aversive effects of blood sampling *^e.g.^* [36], each horse was lightly restrained by one unfamiliar experimenter, gently petted and systematically given a food reward at the end of the sampling (total duration of this procedure <1 min). Four horses became highly agitated when their neck was rubbed with the alcoholic cotton and were not sampled. Seven ml of blood were collected in heparinised polypropylene tubes (BD Vacutainer®). Samples were kept in crushed ice until centrifugation (with a maximal delay between sampling and centrifugation of 15 min) and then aliquots of plasma were immediately placed on dry ice and stored at −20°C for further processing. Blood samples were collected between 18:00 and 19:00 p.m. Two samples were taken for each horse: after a day’s work and after a day’s rest. Plasma cortisol levels were measured using Immunotech kits for cortisol determination (Beckmann and Coulter), usually used for human cortisol plasma determinations and modified so that it could be used for equine plasma (Fureix et al. in prep).

### Data and Statistical Analysis

Analyses were conducted using Statistica© 7.1 software (accepted p level at 0.05). Some differences appeared in horses’ responses between riding schools, but that did not affect the present question and the results, and those data will be published in another paper.

As mentioned above, special attention was given to bouts of immobility when a horse displayed an atypical posture (**Fig.1**) when they seemed to have “withdrawn”. In order to investigate further the features of this spontaneously expressed “withdrawn” posture, gaze durations and ears movements during these bouts were compared to those of four other non-working horses living under natural conditions observed while standing non-resting. The differences in gaze durations and ears movements were however not statistically investigated here due to low number of horses living under natural conditions.

Unresponsiveness to a variety of environmental stimuli, anxiety and cortisol measures were investigated in withdrawn horses by comparing them with non-withdrawn control animals from the same stables. Further analysis including the presence/absence of withdrawn bouts instead of frequencies of occurrence yielded more clear-cut results. Thus, our analysis involved horses displaying the withdrawn posture at least once (called “withdrawn”, or W horses hereafter) and non- withdrawn off horses (never displaying an immobility bout: non-W). Horses’ response to each von Frey stimulation was coded in a binary form (trembling  = 1/not trembling  = 0), that yielded a total tactile reactivity score adding up all a horse’s responses (4 filaments * 3 areas * 2 sides). Further analysis compared horses with “high” tactile reactivity scores (with scores ≥12; 12 being the median score for our sample, *i.e.* the 50% more reactive horses) to horses with “low” tactile reactivity scores (the other 50%). Indifference towards a human was related to the number of C scores in the sudden approach test (see below). Further analysis of the novel object test data compared “highly reactive” horses (*i.e.* with emotionality index ≥38; 38 being the 3^rd^ quartile for our sample, *i.e.* the 25% more anxious horses here) to “less reactive” horses (the other 75%).

As data were not normally distributed, we used non-parametric statistical tests [37]. Chi- square tests compared “withdrawn” to non- withdrawn horses in relation to their breed, sex, riding school and reactions to stimulations (*i.e.* highly reactive or not to tactile stimuli and to the novel object). We used one sample chi-square tests when chi-squared tests of association were impossible because more than 20% of the expected frequencies were below 5 [37]. Mann Whitney tests compared behavioural occurrences, frequencies and cortisol levels between “withdrawn” and “non- withdrawn” horses. Spearman correlation tests correlated frequencies of withdrawn posture and behavioural occurrences (*e.g.* number of “C” responses in the human sudden approach test) and cortisol concentrations. Descriptive statistics are median values (Med), followed by 1^st^ (Q1) and 3^rd^ (Q3) quartiles, range (minimum – maximum).

## Results

### Behavioural Characteristics of “Withdrawn” Horses

In all, 24% of the 59 horses from riding school were observed displaying at least once the withdrawn posture (standing with eyes open, stretched neck, similar height between neck and back) up to 4 times each in 30 minutes. All of these horses were of the same breed (French Saddlebred, SF), which was overrepresented as no horse of another breed presented this syndrome (35% of the SF; 0% of the other breeds, χ^2^_1_ = 8.72, p<0.01). Females were also overrepresented: while one third of the females presented this syndrome (33%, χ^2^_1_ = 1.67, p>0.05), only 20% of the geldings did (χ^2^_1_ = 15.36, p<0.001). Finally, more horses from one riding school presented this syndrome than did horses from the other two schools (school _1_∶0%, school _2_∶45%, school _3_∶6%, χ^2^_2_ = 14.15, p<0.001).

The “withdrawn” posture was also characterized by:

1. The absence of ear and head movements during a bout (that could last from 17 to 97s, Med  = 27.88, Q1 = 22.75, Q3 = 34). This is an unusual trait as horses under natural conditions moved their ears up to 9 times (Med  = 1.23, Q1 = 0.30, Q2 = 3.07, **Fig. 3a**) and their head up to 11 times (Med  = 1.51, Q1 = 0.72, Q2 = 2.16, **Fig. 3b**) during a standing non-resting bout. The ears of all the horses except one (its ears were in an axial position in two of the four withdrawn postures bouts observed for this horse) were directed backwards during a withdrawn posture.
2. A fixed gaze with no eye movements during the whole bout that lasted from 17 to 97 seconds without any visible eye movements (Med  = 27.88, Q1 = 22.75, Q3 = 34)) (median gaze duration while standing non-resting: 15.32, Q1 = 11.95, Q3 = 19.44 under natural conditions, **Fig. 3c**).

### Responsiveness to Environmental Stimuli

#### 1) Tactile responsiveness

**Figure 4:**
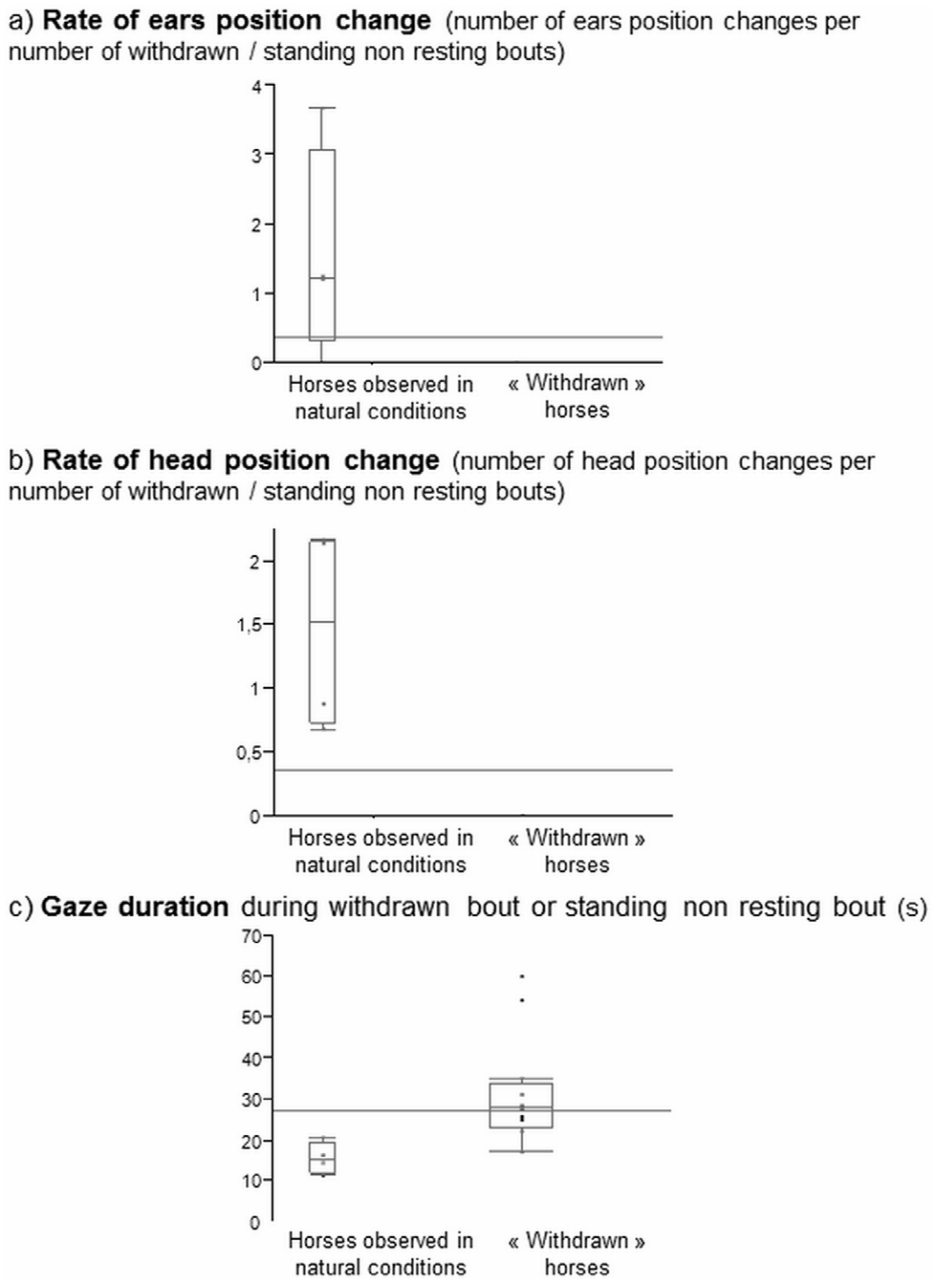
Low responsiveness to environmental stimuli by “withdrawn” horses. (**a**) Proportion of horses with high reactivity scores (*i.e.* ≥12; as 12 is the median score, thus including the 50% more reactive horses) for reactions to von Frey filaments applications, and (**b**) “C” scores (indifference, *i.e.* no change in activity) in the sudden human approach test. Data for (**b**) are given as boxplot diagrams showing medians (lines in the boxes), 25 and 75% quartiles (boxes) and minimum and maximum ranges (whiskers). Only half of the “withdrawn” horses had a high total reactivity score, whereas most of other horses (*i.e.* that never displayed the withdrawn posture) did. “Withdrawn” horses also displayed more often indifference to a sudden human approach than non- withdrawn off horses. Chi-square and Mann Whitney tests, ** p<0.01.

“Withdrawn” horses showed a lowered responsiveness to tactile stimuli. Thus, only half of the “withdrawn” horses (7/14 horses) had a high total reactivity score (*i.e.* a score ≥12, χ^2^_1_ = 0.00, p>0.05), whereas most of the other (*i.e.* that never displayed the withdrawn posture) horses from the same stables had high scores (32/45, χ^2^_1_ = 8.02, p<0.01) (**Fig. 4a**). This was confirmed when concentrating on particular body areas (high tactile reactivity: withers: W horses: 9/14, χ^2^_1_ = 1.14, p>0.05, non-W horses: 36/45, χ^2^_1_ = 16.2, p<0.001; groin: W horses: 9/14, χ^2^_1_ = 1.14, p>0.05, non-W horses: 41/45, χ^2^_1_ = 30.42, p<0.001). Few horses of either category reacted to chest stimulations (W horses: 1/14, χ^2^_1_ = 10.29, p<0.01, non-W horses: 5/45, χ^2^_1_ = 27.22, p<0.001).

#### 2) Reaction to sudden approach of a human

“Withdrawn” horses clearly differed from non-withdrawn horses from the same stables as they generally reacted mostly with indifference (*i.e.* no change in activity) (Mann Whitney test, n _W_  = 14, n _non-W_  = 45, Med_ W_  = 1, Q1 = 0.75, Q3 = 3; Med _non-W_  = 0, Q1 = 0, Q3 = 1; U  = 169.5, p<0.01, **Fig. 4b**). Frequency of no response to the sudden human approach did not differ according to which stable horses were kept (Kruskall-Wallis test, H _(2, N  = 59)_  = 4.67, p>0.05). Interestingly, duration of their withdrawn posture was correlated with the frequency of no response to the approach of a human (Spearman correlation test, N  = 59, r_s__ = _0.36, p  = 0.01).

#### Emotional level/anxiety?

“Withdrawn” horses tented to show higher reactions when confronted with a novel object: 36% (more than one third) of them were in the first reactive quartile of the population (5/14, χ^2^_1_ = 1.14, p>0.05), whereas only 24% of the non-withdrawn horses from the same stables reacted as strongly (11/45, χ^2^_1_ = 11.76, p<0.001).

### Physiological Data: Cortisol Assays

**Figure 5:**
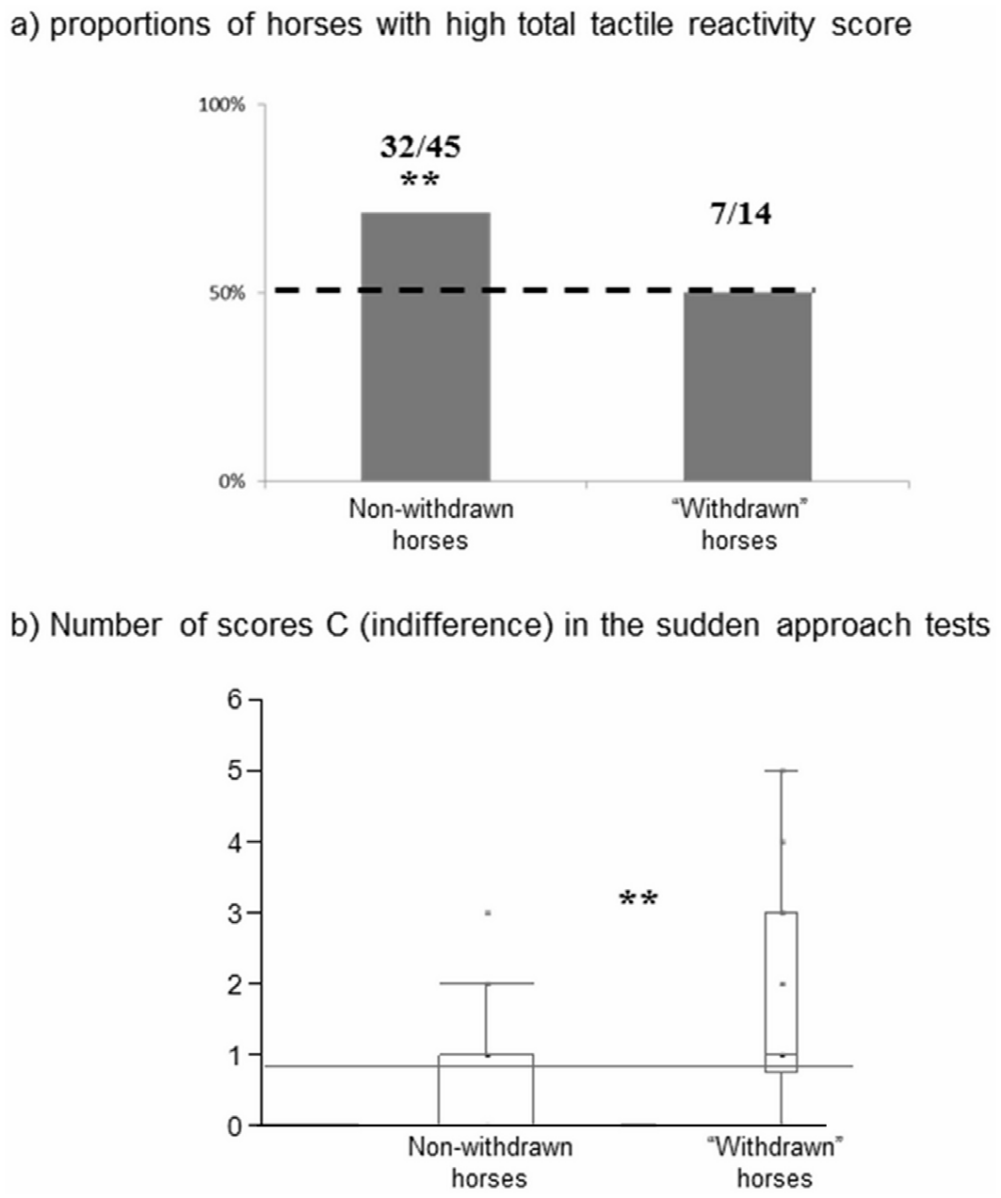
Mean plasma cortisol concentrations after a day’s work for “withdrawn” horses and “non- withdrawn” horses. Data are given as boxplot diagrams showing medians (lines in the boxes), 25 and 75% quartiles (boxes) and minimum and maximum ranges (whiskers). “Withdrawn” horses had lower cortisol concentrations than non- withdrawn horses (*i.e.* that never performed the withdrawn posture). Mann Whitney test, * p<0.05.

Plasma concentrations of cortisol (pC) varied from 2.5 to 57.9 ng/ml after a day’s work and from 3.0 to 35.7 ng/ml after a day’s rest. Interestingly, “withdrawn” horses had lower pC concentrations after a day’s work (Mann Whitney test, n _W_  = 12, n _non-W_  = 43, Med _W_  = 6, Q1 = 6, Q3 = 6.21, Med _non-W_  = 10.65, Q1 = 6, Q3 = 24.09, U  = 144.00, p<0.05) (**Fig. 5**). Moreover, the frequency of withdrawn posture was negatively correlated with pC concentrations after a day’s work (Spearman correlation, N  = 55, r_s_  =  −0.30, p<0.05).

## Discussion

Our observations of horses in their usual domestic environment have led us to spot individuals displaying a particular behavioural and postural profile that presents strong similarities with a “depressive syndrome”. These animals, displaying an atypical posture (stretched neck) and characterized by their unusual gaze, head and ears fixity, were also more indifferent than the others to environmental (tactile and visual) stimuli in their home environment. However, they reacted more emotionally in other more challenging situations. Finally, these “depressed” horses exhibit lower plasma cortisol levels. All these characteristics present strong similarities with some aspect of the depressive states of humans and other animal models *^e.g.^* [4], [6], [7]. Moreover, breed effect (*i.e.* suggested genetic input) and a higher prevalence in females add to the convergence.

Withdrawn horses, like other domestic animals living under unfavourable conditions, showed “apathy” (sows: [15]; horses: [18], [19], “withdrawal” sheep: [16], socially isolated chicks: [38] and lower reactivity to environmental stimulations (pigs: [39], [40]). Pigs submitted to unpredictable electric shocks first become agitated and then inactive [41].

The flatness and fixity of the “withdrawn” horses’ posture when more weight appeared to be put on the fore body and forelegs may reveal chronic pain [42]but also an apparent overloading of the fore body, which evoke the slumped posture of depressive monkeys *^e.g.^* [3], [43]and some depressive patients [44]. The backward ears position suggests discomfort/pain or stress [21]–[23] and altered welfare (Fureix et al. in prep). Decreased eye contact is also observed in depressed patients [5], whereas “withdrawn” horses just gaze “nowhere” and do not attempt to establish eye contact with humans or other horses.

As for human depression, horse “depression” corresponds to a multifaceted syndrome: apathy and loss of interest, lower reactivity but higher anxiety. These horses surprisingly displayed higher emotional responses when facing a challenging situation (novel object in a familiar environment), suggesting, as in depressive humans, a higher level of anxiety *e.g.* [32]. Such emotional reactions have been shown to be influenced by genetic (breed, sire) and environmental (type of work, management) factors in horses *^e.g.^* [34], with high interindividual differences in all horse populations tested *^e.g.^* [30], [33], [45]–[48].

Lower cortisol levels in “withdrawn” horses are in accordance with the results of some studies of depression in communities, which is a more usual environment for humans than hospital settings [8], [10]. Reduced cortisol levels are also observed in chronic fatigue syndrome and posttraumatic stress disorders *^e.g.^* [8], [49], [50] (see also [11] in animals). The lower cortisol levels observed here are also partly in agreement with those observed in other horses submitted to stress-inducing management conditions, presenting lowered cortisol responses in a corticotrophin releasing hormone challenge test [50]. Data for depressive patients are controversial (*e.g.* in [49]) and for some authors, cortisol levels are not a reliable biomarker [6]. However, the lower levels observed here may reflect a depression of the hypothalamo-pituitary-adrenocortical axis, that is, a profound disturbance of the physiological system *^e.g.^* [11], [51], [52].

Our results suggest that horses’ “depressive” states may reflect genetic inputs – as one breed was over represented in our sample – and environmental factors certainly have an effect, as descriptions of “apathetic” horses all correspond to domestic situations when horses experience social, spatial and/or feeding restrictions and potentially stress-inducing work [14], [18], [19], [32]. The prevalence of females displaying this syndrome is another intriguing convergence. These findings suggest that horses as humans and other species may be particularly sensitive to environmental conditions *^e.g.^* [49] that could induce them to develop “depressive syndromes”.

These results open a promising line of investigation of what impaired welfare states could look like in horses. Indeed, though considerable attention has been put on “abnormal” behaviours, *i.e.* stereotypic behaviours (see for instance [53] for a review), little interest has been put on this species on chronic states where horses ‘‘switch off’’, becoming unresponsive and apathetic. Moreover, it has been recently suggested that negative experiences linked to training may add to the effects of management style (*e.g.* social, spatial restrictions) and lead to behavioural despair in horses [18]. Our results suggest that estimations of gaze or body fixity and of body posture might indicate depressive-like state. Adding to the attempts of finding suitable animal welfare indicators, this study would make formally testing the previous hypothesis more feasible.

Beyond direct implications of this work for welfare assessment in horses, horses might be a potential useful candidate to become an animal model for depression, as face validity appears to be high (behavioural similarities) at this stage and construct validity (shared aetiology) may prove fulfilled in future studies when genetic determinism and shared environmental stressors (work constraints, social restriction…) with humans are taken into consideration. Further studies should involve work on anhedonia (loss of pleasure, a core symptom of human depression), cognitive biases (*i.e.* tendency to make negative judgements about events and to interpret ambiguous stimuli unfavourably, typical of depressed humans *^e.g.^* [54], heritability, epidemiology and drugs effects. If confirmed by these studies, horses may well be a particularly useful ethological animal model of human depression. At a time when current animal models of depression are strongly questioned [2], [5], [6]and the need for novel models expressed [2], [7], this study suggests that novel models and biomarkers may well emerge from ethological approaches in the home environment. Estimations of gaze or body fixity and of body posture may reveal to be interesting new biomarkers.
